# Efficacy of Topical Azithromycin versus Systemic Doxycycline in Treatment of Meibomian Gland Dysfunction

**DOI:** 10.1155/2023/4182787

**Published:** 2023-08-08

**Authors:** Marwa Aly Zaky, Adel Galal Zaky, Moataz Fayez Elsawy, Kareem Fatehy Shehata, Mohamed Samy Abd Elaziz

**Affiliations:** ^1^Ophthalmology Department, Faculty of Medicine, Menoufia University, Shebeen El-Kom, Menoufia, Egypt; ^2^Resident at Tanta Ophthalmology Hospital, Tanta, Egypt

## Abstract

**Background:**

Ocular surface disease (OSD) is a multifactorial and highly frequent problem. Inadequate or unstable tear film is the main cause, which leads to visual impairments. One of the primary causes of OSD is meibomian gland dysfunction (MGD), with a prevalence of 3.5 to 70%. The aim of this work was to compare the efficacy of azithromycin topical eye drops versus oral doxycycline in MGD individuals.

**Methods:**

This prospective comparative cohort research was carried out on 56 patients of both sexes of any age with symptomatic MGD. Randomly, patients were classified into two equal groups: Group 1 was treated twice daily for 4 weeks with topical azithromycin 1% eye drops, while group 2 received oral doxycycline 100 mg capsules twice daily for 4 weeks.

**Results:**

In the 1^st^ follow-up, there was a significant difference between the studied groups in pain and discomfort degree (*P* value = 0.024) as group 1 showed a higher number of patients with a mild pain degree (*P* value = 0.013) while group 2 showed a higher number of patients with a severe pain degree (*P* value = 0.022). There was an insignificant difference between the studied groups in moderate pain degree and lid margin telangiectasia. Conjunctivitis, frothy discharge, and meniscus floaters were significantly higher in group 2 than in group 1 (*P* value = 0.013, 0.028, and 0.031, respectively). In group 1, the break-up time test was significantly higher than in group 2 (*P* value = 0.023). In the 2^nd^ follow up, in group 2 only meniscus floaters were significantly higher than in group 1 (*P* value = 0.044), while in group 1 break-up time test was significantly higher than in group 2 (*P* value = 0.029). Otherwise, there is no significant difference between both the groups.

**Conclusions:**

Meibomian gland dysfunction (MGD) could be treated effectively with oral doxycycline and topical azithromycin by improving symptoms, clinical signs, and stabilization of tear film. Moreover, the topical azithromycin group seemed to be superior over the oral doxycycline group in improving the quality of tear film in the short term, having fewer side effects, more compliance, and better tolerability.

## 1. Introduction

Ocular surface disease (OSD) is a multifactorial and a highly frequent problem which may lead to pain, inflammation, irritation, photophobia, blurred vision, and vision loss [1]. There are many essential causes of OSD such as inadequate or unstable tear film and neurotrophic keratopathy (NK) which is a degenerative corneal disorder that affects the health and integrity of the ocular surface secondary to impairment of corneal nerves that alters their trophic and sensory function [2]. Also, meibomian gland dysfunction (MGD) incidence varies widely from 3.5 to 70% based on ethnicity, sex, and age. MGD also has a significant influence on the patients' life quality, as it is frequently a contributing factor to evaporative dry eye and is responsible for symptoms of irritation of the eyelid and ocular surface. [3].

Risk factors of MGD include aging, deficiency of sex hormones specially androgens, other systemic diseases such as Stevens–Johnson syndrome (SJS), atopy, Sjogren's syndrome (SS), psoriasis, and hypertension. Also, ophthalmic disorders such as chronic blepharitis, contact lens wear, and trachoma may play a role. Demodex folliculorum infestation and aniridia have been shown to affect meibomian gland function. The use of antibiotics, isotretinoin for acne, antihistamines, antidepressants, and hormone replacement therapy has been found to be associated with MGD [3, 4].

MGD pathophysiology is caused by obstruction of meibomian gland and hyper-keratinization of the epithelium of the meibomian duct which results in decreased meibum availability over the tear film aqueous layer. Reduced meibum leads to elevation of bacterial growth on the margin of the lid, instability of the tear film, increased hyperosmolarity, and tears evaporation [4]. MGD choices of treatment vary from initial conservative interventions involving eyelid massage, eyelid expression, and warm compression to, in more severe cases, medical intervention with anti-inflammatory drugs. In past trials, antibiotics such as doxycycline and azithromycin were proven to effectively treat both the signs and symptoms of MGD patients [5].

Doxycycline is one of the standard treatments for MGD, which is a derivative of tetracycline and has been used because of its lipid-regulating, anti-inflammatory, and antimicrobial characteristics. It suppresses matrix metalloproteinases, which are responsible for connective tissue degradation. It has been used to treat ocular rosacea by alleviating symptoms of irritation and increasing the stability of the tear film. In addition, it has been used to cure erosions of the cornea [6].

Topical azithromycin has been identified as a potentially well-tolerated and effective treatment for MGD and diseases of the lid margin in clinical trials. Azithromycin is a macrolide antibiotic that has a broad-spectrum action, anti-inflammatory characteristics, and a lipid-regulating effect. In addition, at concentrations achieved by topical administration, it displayed bacteriostatic activity, resulting in the bacterial growth suppression on the lid margin [7].

This work aimed to compare topical azithromycin eye drops versus oral doxycycline efficacy in MGD patients.

## 2. Patients and Methods

This comparative prospective cohort study was carried out on 56 patients of both the sexes, of any age who presented with symptomatic MGD. It was done in the Ophthalmology Department Menoufia University Hospital from September 2020 to September 2021.

An informed written consent was obtained from the patient or relatives of the patient. The study was done after approval from the Ethics Committee Menoufia University Hospitals (19819OPHT23).

Exclusion criteria were history of any ocular surface surgery (including pterygium resection and refractive surgeries), ocular injuries, pregnant and lactating women, women on oral contraceptive pills, a known hypersensitivity to azithromycin or doxycycline, contact lens wearing, gross lid structural abnormality, and corneal scarring. The use of any of the following medications within one month of the study: ocular or oral antibiotics, topical or systemic steroids, ocular nonsteroidal anti-inflammatory drugs, topical cyclosporine, topical antihistamine, and/or mast cell stabilizers.

All patients were subjected to personal history taking and a thorough ophthalmic examination involving (measurement of visual acuity, manifest refraction, and slit lamp biomicroscopy).

Patients with MGD were randomly (by computer-assisted program) and equally divided into two groups. Group 1 was treated twice daily for 4 weeks with topical Cyanaro (azithromycin 1%) eye drops (Talent Pharma Group, New Cairo, Egypt) and group 2 received oral vibramycin (100 mg doxycycline) capsules (Pfizer, Cairo, Egypt) twice daily for 4 weeks. The two groups were maintained on conservative management in the form of eyelid massage, cleaning using eye-friendly soap at bedtime (Baby Johnson Shampoo, Johnson & Johnson), warm fomentation twice daily, and artificial tear eye drop Systane Ultra (Alcon Laboratories, Fort Worth, Texas, USA) 4 times daily. The data were collected: On admission, after 2 weeks (first follow-up) and after 4 weeks (second follow-up), and the parameters were compared between both groups.

Pain assessment was done according to the Wong and Baker Face Pain Scale which was created by Donna Wong and Connie Baker. The scale depicts a faces sequence fluctuating from a crying face at 10, which indicates “hurts like the worst pain imaginable to a happy face at 0, which indicates “no hurt.” According to the written descriptions and faces, the patient chooses the face that most closely fits their pain level. In our study, the Wong and Baker pain scale was categorized into three degrees: (severe pain: 7–10, moderate pain: 4–6, and mild pain: 1–3).

As regards the lid margin telangiectasia scale, the grading scale is dependent on definite properties. Abnormal vascularity findings of the lid margin are dependent on 2 major components: the distribution of telangiectasia crossing meibomian gland orifices and the lid margin redness degree.

0 = no or slight redness in the lid margin conjunctiva and no telangiectasia crossing meibomian gland orifices.

1 = redness in the lid margin conjunctiva and no telangiectasia crossing meibomian gland orifices.

2 = redness in the lid margin conjunctiva and telangiectasia crossing meibomian gland orifices with a distribution of less than half of the full length of the lid.

3 = redness in the lid margin conjunctiva and telangiectasia crossing meibomian gland orifices with a distribution of half or more of the full length of the lid.

Also, we assessed the presence or absence (positive or negative) of conjunctivitis, frothy discharge, and tear film meniscus floaters.

Break-up Time Test: It is a clinical examination that is used to diagnose evaporative dry eye disease. The patient's tear film is instilled with fluorescein for TBUT measurement, and the patient is urged not to blink while the tear film is detected by a broad beam of cobalt blue illumination.

Following a time interval, lines or black spots appear in the fluorescein-stained film, indicating the establishment of dry patches. The time interval between the final blink and the emergence of the first dry spot is referred to as the TBUT. A TBUT shorter than ten seconds is seen as abnormal.

### 2.1. Statistical Analysis

SPSS v26 (IBM Inc., Chicago, IL, USA) was used to perform statistical analysis. Quantitative variables were presented as the mean and standard deviation (SD) and utilize unpaired Student's t-test to compare the two groups. Qualitative data were presented as frequency and percentage (%), and chi-square test or Fisher's exact test when appropriate were used in their analysis. The level of significance for all tests was set at *P* < 0.05.

## 3. Results

There was an insignificant difference between both groups in age and gender. (Table 1) Pain and discomfort, lid margin telangiectasia, conjunctivitis, frothy discharge, meniscus floaters, and break-up time tests in the 1^st^ visit were insignificantly different between both groups. (Table 2)

In the 1^st^ follow-up, there was a significant difference between the studied groups in pain and discomfort degree (*P* value = 0.024). Patients with mild pain degrees were significantly higher in the topical azithromycin group (*P* value = 0.013), and patients with severe pain were significantly higher in the systemic doxycycline group (*P* = 0.022). There was an insignificant difference between the studied groups in moderate pain degree and lid margin telangiectasia. Conjunctivitis and meniscus floaters were significantly lower in the topical azithromycin group than in the systemic doxycycline group (*P* value = 0.013, 0.031, respectively). Break-up time tests and patients with negative frothy discharge were significantly higher in the topical azithromycin group than in the systemic doxycycline group (*P*=0.023, 0.028, respectively). (Table 3)

In the 2^nd^ follow-up, pain and discomfort, lid margin telangiectasia, conjunctivitis, and frothy discharge were insignificantly different between the studied groups. Meniscus floaters in the 2^nd^ follow-up were significantly lower in the topical azithromycin group than in the systemic doxycycline group (*P* value = 0.044), while break-up time test was significantly higher in the topical azithromycin group (*P* value = 0.029). (Table 4)

With regards to side effects in both groups, burning sensation was significantly higher in the topical azithromycin group (*P*=0.023) and GIT upset was significantly higher in the systemic doxycycline group (*P* < 0.001). Absence of any side effects, stinging sensation, sticky eyelids, photosensitivity, and allergic reaction were insignificantly different between the two groups.

## 4. Discussion

Obstructive MGD is the most prevalent cause of EDE and occurs as a primary disorder or secondary to seborrheic or atopic dermatitis, acne rosacea, and cicatrizing conjunctival disorders, such as erythema multiforme, trachoma, and cicatricial pemphigoid [8].

The main goal of the treatment of MGD is to improve the quality and quantity of meibomian glands secretion and thus relieve discomfort. Many studies have shown the essential role of eyelid warming and massaging and topical antibiotics such as macrolides and tetracycline in the treatment of MGD. Recent studies demonstrated intense pulsed light (IPL) efficacy can enhance manifestations in patients with dry eye disorder (DED) and MGD. However, the exact mechanisms of action are still not completely clear, the thermal effect produced by a series of pulses of noncoherent polychromatic light results in the liquefaction of meibum, reducing proinflammatory mediators and matrix metalloproteinases' production. Furthermore, low-level light therapy (LLLT), using light-emitting diodes (LEDs) at wavelengths insufficient to create a thermal effect, has shown good results in dermatology through a mechanism of action known as photobiomodulation [9].

Antibiotics such as lymecycline and azithromycin all help reduce dry eye symptoms because of their anti-inflammatory action. There are a surprisingly large number of antibiotics that have a concomitant anti-inflammatory action which we can use as an important treatment for dry eye disease due to meibomian gland dysfunction. The action is twofold in that they alter, reduce, or alter the eyelid flora, and the microbiome that is contributing to the meibomian gland dysfunction alters the ocular surface inflammation through their direct anti-inflammatory effect. Full doses do not have to be taken, and small doses can be used for a longer period of time. The two main groups of antibiotics that we use are tetracyclines and macrolides. Oral doxycycline, minocycline, or lymecycline successfully improve symptoms and signs of meibomian gland dysfunction. However, there are some potential gastrointestinal side effects. There is also some concern about whether they will affect the gut microbiome. For instance, it is not totally clear what long-term effects doxycycline or lymecycline have on altering the gut flora and how, therefore, the gut microbiome may modulate the body's immune system. An alternative is to use azithromycin either topically, orally, or both [5, 8].

Our study assessed the efficacy of topical azithromycin versus systemic doxycycline in the treatment of meibomian gland dysfunction. We reported insignificant different results regarding the pain and discomfort, lid margin telangiectasia, and conjunctivitis between both studied groups in the first visit.

Regarding pain and discomfort, a long-term, randomized study conducted by De Benedetti and Vaiano [8] compared doxycycline (4 g for 30 days) and oral azithromycin (1.25 g for 5 days) in MGD patients for 9 months and discovered comparable results to ours as both groups showed an insignificant difference in pain degree in the first visit. They also reported that there was no significant difference between the doxycycline and azithromycin group in conjunctivitis. Concerning the lid margin telangiectasia, our results are similar to those of Foulks et al. [10] who demonstrated that there was no significant difference was recorded between the doxycycline and azithromycin group regarding lid margin telangiectasia.

In addition, the present results revealed that there was no significant difference observed between the azithromycin and doxycycline groups with respect to frothy discharge and meniscus floaters in the first visit. Our findings are in accordance with Hamed et al. [11] who assessed their comparative study to compare the outcome of using topical azithromycin and doxycycline in improving signs and symptoms of posterior blepharitis causing MGD. One hundred and twenty eyes of sixty patients of both sexes above the age of 18 diagnosed with posterior blepharitis causing dry eye disease were enrolled. The results showed that in the first visit, the frothy discharge between groups was insignificantly different.

Moreover, concerning the first visit, the break-up time test was insignificantly different between both groups. Our results agree with Foulks et al. [10] who reported that the break-up time of tear of patients with MGD was statistically nonsignificant between azithromycin and doxycycline groups in admission time.

Our results demonstrated that in the first follow-up there was a significant difference between the studied groups in pain and discomfort degrees. The number of patients with mild pain degrees was significantly higher in the group of topical azithromycin and the number of patients with severe pain was significantly higher in the systemic doxycycline group. There was an insignificant difference between the studied groups in moderate pain degree. Similarly, Kashkouli et al. [5] showed that signs and symptoms in both groups had improved after 60 days of treatment, but the overall symptoms in the azithromycin group, including pain degree and signs, decreased to a mild score. The reduction in symptoms, pain degree, and signs was more significant in the azithromycin group compared to the doxycycline group.

Furthermore, our study demonstrated that the lid margin telangiectasia in 1^st^ follow-up was insignificantly different between the studied groups. Our results are against Kashkouli et al. [5] who found that in the first follow-up of patients, there was a significant decrease in lid margin telangiectasia in the azithromycin group compared to the doxycycline group. The limited sample size of our study may account for this difference.

Besides, the obtained results explained that the conjunctivitis in 1^st^ follow-up after 2 weeks of treatment was significantly lower in the topical azithromycin 6 (21.43%) than systemic doxycycline 15 (53.57%). The results recorded by Foulks et al. [10] matched with ours where the conjunctivitis in 1^st^ follow-up after 1 week was significantly lower in topical azithromycin 1.10 ± 0.88 than systemic doxycycline 1.57 ± 0.73.

Moreover, our recorded data showed that in the 1^st^ follow-up, patients with negative frothy discharge were significantly higher in the topical azithromycin group than in the systemic doxycycline group. Our results disagree with Hamed et al. [11] who demonstrated that the signs of 1^st^ follow-up after 1 week showed a nonsignificant difference between the azithromycin group and the doxycycline group in frothy discharge. The short follow-up and the different sample sizes led to the difference in outcomes between our and Hamed et al. studies.

Furthermore, the meniscus floaters in 1^st^ follow-up of our findings were significantly lower in topical azithromycin than systemic doxycycline.

Similar results were reported by Lam et al. [12] who conducted, in 2020, a systematic review of the recent publications concerning MGD treatment options. A total of 35 articles were examined for relevance. Generally, all treatment modalities were clinically efficient in reducing dry eye signs and symptoms; however, the degree of improvement and persistence of effects varied between the different treatments.

In addition, our results showed that in the 1^st^ follow-up, the break-up time test was significantly higher in the topical azithromycin group. These results are in agreement with Hamed et al. [11] who demonstrated that in the first follow-up for the azithromycin group and the doxycycline group the tear break-up time was significantly increased in azithromycin-treated patients compared to the doxycycline group.

Besides, the present research results illustrated that pain and discomfort, lid margin telangiectasia, and conjunctivitis in the 2^nd^ follow-up were insignificantly different between studied groups.

These findings are comparable to those of Satitpitakul et al. [13] who carried out a prospective randomized trial to evaluate the efficacy of azithromycin 1.5% eyedrops in individuals with moderate to severe MGD when compared to oral doxycycline. Newly diagnosed moderate-to-severe MGD in 169 cases were involved. 85 patients were randomly assigned to receive azithromycin 1.5% eyedrops twice daily for two days and then once daily for four weeks, and 84 patients received oral doxycycline 100 mg twice daily for four weeks. Symptoms and signs of MGD were assessed later at the baseline and after 4 weeks. The results explained that after 4 weeks of follow-up, MGD-related symptoms including discomfort symptom was shown in 54 (75.00%) patients in the azithromycin group compared to 55 (78.57%) patients in the doxycycline group.

Moreover, Foulks et al. [10] reported a similar result: all signs of the disease of the eyelid margin including telangiectasia alleviated after 4 weeks follow-up in both groups with no statistically significant difference between azithromycin and doxycycline groups.

In addition, De Benedetti and Vaiano [8] showed a similar result in 2^nd^ follow-up as there was no statistically significant difference between both groups concerning the conjunctivitis.

Likewise, another major finding in the second follow-up was that the frothy discharge was insignificantly different between the investigated groups.

Moreover, this result was consistent with Fadlallah and colleagues [14] who carried out a study to evaluate topical 1.5% azithromycin efficacy in chronic blepharitis of moderate-to-severe degree treatment and to compare the efficacy of two different treatment modalities. 67 patients with chronic anterior and/or posterior blepharitis resulting in dry eyes were included in a randomised clinical trial and observed for three months. Symptoms and signs were graded based on the severity. Patients were randomly assigned into two groups: Group I received for three days 1.5% azithromycin topically twice daily; Group II received 1.5% azithromycin topically twice daily for three days followed by bedtime treatment for the remainder of the month.

The results showed insignificant improvement in frothy discharge, and this may be due to the fact that azithromycin could not reach the therapeutic level on the meibomian gland to stabilize the tear film.

Interestingly, our results indicated that in the 2^nd^ follow-up, the break-up time test was significantly higher in the topical azithromycin group. These results matched with those of Foulks et al., [10] who demonstrated that in the 2^nd^ follow-up after 4 weeks for azithromycin-treated patients and 8 weeks for doxycycline treated patients, the tear break-up time was significantly higher in patients treated with azithromycin compared to the doxycycline group.

Also, a better understanding of healthy eye microbiota has the potential to improve disease diagnosis and personalized regimens to promote health. [15].

### 4.1. Limitations

The sample size of this study was relatively small, there was a short follow-up period, and it was a single-centric study. Also, we did not conduct the various tests of visual function such as contrast sensitivity test and colour vision tests.

## 5. Conclusions

Meibomian gland dysfunction (MGD) could be treated effectively with oral doxycycline and topical azithromycin by improving symptoms, clinical signs, and stabilization of the tear film. Moreover, topical group seemed to be superior over oral group in improving the quality of the tear film in the short term, with fewer side effects, more compliance, and better tolerability.

## Figures and Tables

**Table 1:** Demographic data of the studied groups.

Age (years)		Topical azithromycin (*n* = 28)	Systemic doxycycline (*n* = 28)	*P* value
		30.11 ± 12.12	35.00 ± 14.14	0.170
		15–56	16–60	
Gender	Male	16 (57.14%)	18 (64.29%)	0.584
	Female	12 (42.86%)	10 (35.71%)	

Data are presented as mean ± SD.

**Table 2:** Pain and discomfort, lid margin telangiectasia, conjunctivitis, frothy discharge, meniscus floaters and break-up time test in 1^ry^ visit between studied groups.

		Topical azithromycin (*n* = 28)	Systemic doxycycline (*n* = 28)	*P* value
Pain and discomfort	Mild	6 (21.43%)	5 (17.86%)	0.248
	Moderate	12 (42.86%)	7 (25.00%)	
	Severe	10 (35.71%)	16 (57.14%)	

Lid margin telangiectasia	0 & 1	1 (3.57%)	2 (7.14%)	0.661
	2	7 (25.00%)	9 (32.14%)	
	3	20 (71.43%)	17 (60.71%)	

Conjunctivitis	+ve	16 (57.14%)	19 (57.86%)	0.407
	−ve	12 (42.86%)	9 (32.14%)	

Frothy discharge	+ve	25 (89.28%)	21 (75%)	0.295
	−ve	3 (10.71%)	7 (25%)	

Meniscus floaters	+ve	21 (75.00%)	22 (78.57%)	0.751
	−ve	7 (25.00%)	6 (21.43%)	

Break-up time test (seconds)		6.25 ± 2.07	6.54 ± 1.88	0.590
		2–9	3–9	

Data are presented as mean ± SD.

**Table 3:** Pain and discomfort, lid margin telangiectasia, conjunctivitis, frothy discharge, meniscus floaters and break-up time test in 1^st^ follow up between studied groups.

		Topical azithromycin (*n* = 28)	Systemic doxycycline (*n* = 28)	*P* value	
Pain and discomfort	Mild	15 (53.57%)	6 (21.43%)	0.024^*∗*^	0.013^*∗*^
	Moderate	8 (28.57%)	9 (32.14%)		0.771
	Severe	5 (17.86%)	13 (46.43%)		0.022^*∗*^

Lid margin telangiectasia	0 & 1	4 (14.29%)	3 (10.71%)	0.563	
	2	13 (46.43%)	10 (35.71%)		
	3	11(39.29%)	15 (53.57%)		

Conjunctivitis	+ve	6 (21.43%)	15 (53.57%)	0.013^*∗*^	
	−ve	22 (78.57%)	13 (46.43%)		

Frothy discharge	+ve	7 (25%)	16 (57.14%)	0.028^*∗*^	
	−ve	21 (75%)	12 (42.86%)		

Meniscus floaters	+ve	10(35.71%)	19(67.86%)	0.031^*∗*^	
	−ve	18(64.29%)	9(32.14%)		

Break-up time test (seconds)		7.93 ± 1.94	6.61 ± 2.27	0.023^*∗*^	
		5–13	3–11		

Data are presented as mean ± SD, ^*∗*^: significant as *P* value ≤ 0.05.

**Table 4:** Pain and discomfort, lid margin telangiectasia, conjunctivitis, frothy discharge, meniscus floaters and break-up time test in 2^nd^ follow up between studied groups.

		Topical azithromycin (*n* = 28)	Systemic doxycycline (*n* = 28)	*P* value
Pain and discomfort	Mild	19 (67.86%)	17 (60.71%)	0.828
	Moderate	7 (25.00%)	8 (28.57%)	
	Severe	2 (7.14%)	3 (10.71%)	

Lid margin telangiectasia	0 & 1	13 (46.43%)	9 (32.14%)	0.452
	2	11 (39.29%)	12 (42.86%)	
	3	4 (14.29%)	7 (25.00%)	

Conjunctivitis	+ve	4 (14.29%)	11 (39.29%)	0.068
	−ve	24 (85.71%)	17 (60.70%)	

Frothy discharge	+ve	1 (3.57%)	3 (10.71%)	0.611
	−ve	27 (96.43%)	25 (89.28%)	

Meniscus floaters	+ve	5 (17.86%)	13 (46.43%)	0.044^*∗*^
	−ve	23 (82.14%)	15 (53.57%)	

Break-up time test (seconds)		9.11 ± 2.64	7.71 ± 1.98	0.029^*∗*^
		6–14	5–11	

Data are presented as mean ± SD, ^*∗*^: significant as *P* value ≤ 0.05.

## Data Availability

The datasets used and/or analyzed during the current study are available from the corresponding author on reasonable request.
